# Age-related differences in facial emotion recognition across adulthood: accuracy and confidence in the 30s, 50s, and 70s

**DOI:** 10.3389/fpsyg.2026.1859795

**Published:** 2026-09-03

**Authors:** Midori Tokita, Sawako Sato

**Affiliations:** Department of Health Sciences, Mejiro University, Saitama City, Saitama, Japan

**Keywords:** adulthood, age differences, confidence judgment, empathy, facial emotion recognition, gender differences, metacognition

## Abstract

**Introduction:**

Age-related changes in facial emotion recognition have been widely documented; however, relatively few studies have examined these changes across adulthood, including middle age. Little is also known about how confidence in these judgments changes across adulthood and how these processes relate to empathy. The present study examined age and gender differences in recognition accuracy, confidence, and empathy, as well as the relationships among these factors.

**Methods:**

A total of 695 adults in their 30s, 50s, and 70s were included in the final analyses. Participants completed a facial emotion recognition task with six emotional expressions and provided confidence ratings for each response. They also completed a self-report measure of cognitive and affective empathy.

**Results:**

Age group differences in recognition accuracy were emotion-specific and, in some cases, modulated by gender. Confidence analyses showed that age group was associated with an increased likelihood of high-confidence errors, whereas no significant age-group effect was observed for high-confidence correct responses; a direct test of the age group × correctness interaction did not reach significance. Empathy analyses indicated small but consistent gender differences, with women scoring higher than men, and no clear age-related effects. Associations between empathy and recognition performance were modest, with cognitive empathy showing a small positive relationship in men aged in their 70s, whereas affective empathy showed no clear association.

**Discussion:**

These findings indicate that age group differences in facial emotion recognition involve not only perceptual accuracy but also metacognitive evaluation, and do not support a simple decline in performance in later adulthood. The results further suggest that cognitive and affective empathy differ in their relationship to emotion recognition, indicating partially distinct components of socioemotional functioning across adulthood.

## 1 Introduction

Adaptive social behavior relies on the efficient processing and interpretation of facial emotional cues. Misreading emotional signals, such as failing to recognize subtle fears, discomfort, or uneasiness, can lead to responses that are mismatched to the social context (van Kleef and Côté, 2022). Effective interpersonal behavior depends not only on the accurate perception of others’ emotional states but also on the appropriate evaluation of one’s own interpretations (Bègue et al., 2019). From an experimental perspective, facial emotion recognition can be conceptualized as a form of social perceptual processing supported by underlying perceptual-cognitive mechanisms and accompanied by metacognitive monitoring (Kelly and Metcalfe, 2011).

Substantial research has demonstrated age-related differences in facial emotion recognition. Meta-analytic evidence suggests that older adults often exhibit lower recognition accuracy than younger adults (Olderbak et al., 2019; Ruffman et al., 2008). However, these age-related differences are not uniform across emotional categories. The recognition of negative emotions, such as fear and anger, tends to decline more consistently, whereas the recognition of happiness is frequently preserved (Isaacowitz and Stanley, 2011; Mill et al., 2009). Similar patterns have been observed for both static facial expressions and dynamic emotional expressions (Abiodun et al., 2024; Hayes et al., 2020). In addition, gender differences exist, with women often outperforming men, particularly in the recognition of socially salient negative expressions (Kret and De Gelder, 2012; McClure, 2000). These findings indicate that age- and gender-related variations in emotion recognition likely reflect the selective modulation of specific perceptual-cognitive processes rather than a generalized decline (Ruffman et al., 2008). Additionally, age-related differences may be more pronounced for subtle or low-intensity facial expressions than for highly salient ones (Mienaltowski et al., 2019; Orgeta and Phillips, 2008). Low-intensity expressions require finer perceptual discrimination and the integration of multiple facial cues (Calder et al., 2000), which have been proposed to change with age, particularly with respect to the precision of processing individual facial features (Meinhardt-Injac et al., 2014; Fry et al., 2023). Accordingly, ambiguous expressions may be particularly informative when examining potential nonlinear patterns across adulthood.

However, several limitations remain in the literature. First, much of the literature relies on comparisons between young adults and adults aged 60 years or older, and few studies include middle-aged participants (Ebner et al., 2010; Mill et al., 2009), although some recent work has begun to address this gap (Faustmann et al., 2022). Consequently, midlife has been relatively under-examined, leading to a lack of clarity regarding nonlinear or midlife-specific patterns (Isaacowitz and Stanley, 2011). Mill et al. (2009) examined age-related differences in emotion recognition across a broad adult age range and reported that recognition accuracy declines with increasing age, particularly for negative emotions. However, gender differences were not a focus of that study. From the perspective of lifespan cognitive development, experience-related changes during middle adulthood may not follow a simple linear decline (Baltes and Baltes, 1990; Blanchard-Fields, 2007; Charles, 2010). Accumulated interpersonal experience, occupational demands, and repeated exposure to diverse social situations, including gender-typed societal roles, may contribute to the maintenance or optimisation of specific forms of social perceptual processing during midlife. This process may enhance sensitivity to particular emotional cues before more pronounced neural and cognitive changes occur later in life (Lin et al., 2024). Supporting this possibility, certain socio-cognitive traits, such as aspects of empathy, demonstrate nonlinear patterns across adulthood (Grühn et al., 2008). These findings suggest that facial emotion recognition may also follow non-monotonic developmental trajectories.

Second, most previous studies have focused exclusively on first-order accuracy, treating emotion recognition as a perceptual categorization task. However, perceptual decisions are accompanied by metacognitive evaluations (Fleming and Daw, 2017). Confidence judgments provide information on how individuals monitor and assess the reliability of their responses. Research on memory and perceptual decision-making has shown that aging may be associated with reduced calibration between confidence and objective accuracy, often expressed as increased overconfidence in errors (Dodson et al., 2007; Palmer et al., 2014). Whether similar age-related changes occur during emotional facial processing remains unclear. Investigating confidence in emotion recognition enables examination of second-order perceptual monitoring mechanisms that may follow developmental trajectories distinct from those of first-order accuracy.

Empathy is another construct relevant to emotion recognition. Empathy is typically conceptualized as comprising cognitive and affective components (Brett et al., 2023; Davis, 1983; Decety and Jackson, 2004). Cognitive empathy refers to perspective-taking and the inference of others’ mental states, whereas affective empathy involves emotional responsiveness to others’ feelings. Prior research on empathy has documented gender differences but suggests mixed findings regarding age-related changes (Grühn et al., 2008; O’Brien et al., 2013). O’Brien et al. (2013) demonstrated that empathy may follow an inverted U-shaped trajectory throughout adulthood, with relatively higher levels in midlife. A recent meta-analysis found that older adults show modestly higher emotional empathy than younger adults, although the direction and magnitude of this effect varied across specific measures (Jarvis et al., 2024). Using ecological momentary assessment, Pollerhoff et al. (2022) similarly found empathy to increase from young adulthood into midlife before showing a slight decline in adults aged 55 and older, suggesting this nonlinear pattern may extend to real-life empathic experience. From a perceptual-cognitive standpoint, individuals with higher empathy may be more sensitive to emotional cues, potentially enhancing their emotion recognition performance (Besel and Yuille, 2010; Zaki, 2014). However, the relationship between emotion recognition and both cognitive and affective empathy is ambiguous (Moret-Tatay et al., 2023), and empirical findings regarding the strength and developmental stability of the empathy–recognition association are inconsistent (Olderbak and Wilhelm, 2017). Whether this relationship is stable throughout adulthood or varies with age and gender remains unclear (Beadle and de la Vega, 2019).

Thus, several questions remain unanswered. How does facial emotion recognition change during midlife? Do gender differences persist or vary throughout adulthood? Does aging influence metacognitive calibration in emotion recognition, particularly in the form of high-confidence errors? Moreover, how are cognitive and affective empathy associated with individual differences in emotional face processing across the adult lifespan?

The primary objective of this research is to characterize the developmental trajectory of emotional face recognition from middle to late adulthood. Due to the current lack of longitudinal data spanning these decades, the present study initiates this investigation by examining cross-sectional age-group differences among adults in their 30s, 50s, and 70s, including both men and women. The inclusion of middle-aged participants extends the analysis beyond a simple young versus old comparison and allows for the assessment of whether age-group patterns across adulthood align with nonlinear, experience-related changes.

In addition to the conventional recognition accuracy, confidence ratings were obtained after each emotion judgment to assess the metacognitive aspects of perceptual performance. This approach enables dissociation between changes in perceptual sensitivity and changes in monitoring or calibration processes. Empathy was assessed using the Perth Empathy Scale (PES), which distinguishes cognitive and affective components within a unified measurement framework (Brett et al., 2023; Ye et al., 2024). Assessing these components separately enables a more precise examination of whether distinct aspects of empathy are differentially related to emotion recognition and whether these associations vary across age and gender groups.

The study also incorporated methodological refinements to enhance the sensitivity to subtle age- and gender-related differences. Prior research has noted that certain facial expressions, particularly happiness, are often recognized with near-ceiling accuracy, potentially limiting the sensitivity to individual or age-related differences (Calder et al., 2000; Ruffman et al., 2008). Emotional intensity was systematically controlled using morphing procedures that blended neutral and emotional expressions at predefined ratios (Kumada et al., 2011), thereby standardizing the task difficulty across categories. In addition, diagnostic facial cues were reduced for selected expressions to minimize ceiling effects commonly observed in happiness recognition. By equating stimulus difficulty and reducing reliance on salient cues, the design aimed to isolate differences in perceptual-cognitive processing rather than the artifacts of task simplicity.

Based on these considerations, we hypothesized the following. First, we expected age- and gender-related differences in emotion recognition accuracy with variations across emotional categories. Considering the limited prior evidence concerning midlife, we examined whether performance in the 50s would be maintained or potentially enhanced relative to that in the 30s, enabling the examination of nonlinear age-related patterns. Second, drawing on metacognitive research, we predicted that older age would be associated with an increased likelihood of high-confidence errors, reflecting a reduced calibration between confidence and accuracy. Third, we expected women to report higher levels of empathy than men, particularly for affective empathy, and examined whether empathy scores would show nonlinear variation across adulthood. Finally, we examined whether empathy is associated with emotion recognition performance and whether this relationship varies across age and gender.

By integrating age, gender, emotional face processing, metacognitive evaluation, and distinct components of empathy within a single experimental framework, the present study sought to clarify how the perceptual-cognitive mechanisms underlying emotion recognition change across adulthood.

## 2 Methods

### 2.1 Participants

Participants were recruited through an online research panel managed by Cross Marketing, Inc., a Japanese survey research company. Panel members who met the target age groups were invited to participate in the study; no information was collected regarding mental, neurological or overall health. A total of 948 adults participated in the study. The sample consisted of men and women in their 30s, 50s, and 70s: 150 men in their 30s, 166 in their 50s, and 173 in their 70s, and 150 women in their 30s, 159 in their 50s, and 150 in their 70s. Participants self-reported their gender as male or female. As the survey did not explicitly distinguish between sex assigned at birth and gender identity, the term ‘gender’ is used consistently throughout this manuscript. After applying the predefined exclusion criteria (Section 2.4), the final sample consisted of 695 adults; the age and gender distributions are shown in Table 1.

**Table 1:** Description of the analytic sample.

	30s (*n* = 191)		50s (*n* = 237)		70s (*n* = 267)	
	Women (*n* = 101)	Men (*n* = 90)	Women (*n* = 119)	Men (*n* = 118)	Women (*n* = 122)	Men (*n* = 145)
Mean age (years)	35.0	35.5	54.0	54.5	73.5	73.3
SD of Age (years)	2.65	2.73	2.86	2.69	2.54	2.43

All participants were native Japanese speakers residing in Japan. The participants completed the experiment using smartphones or tablets. The participants were allowed to complete the tasks at their own pace. All participants provided informed consent electronically prior to participation.

### 2.2 Materials and procedures

The survey consisted of two parts: an emotion recognition task using facial stimuli and the PES. Based on a preliminary study, the expected time to complete the survey was approximately 6–15 min.

#### 2.2.1 Emotion recognition task

**Figure 1:**
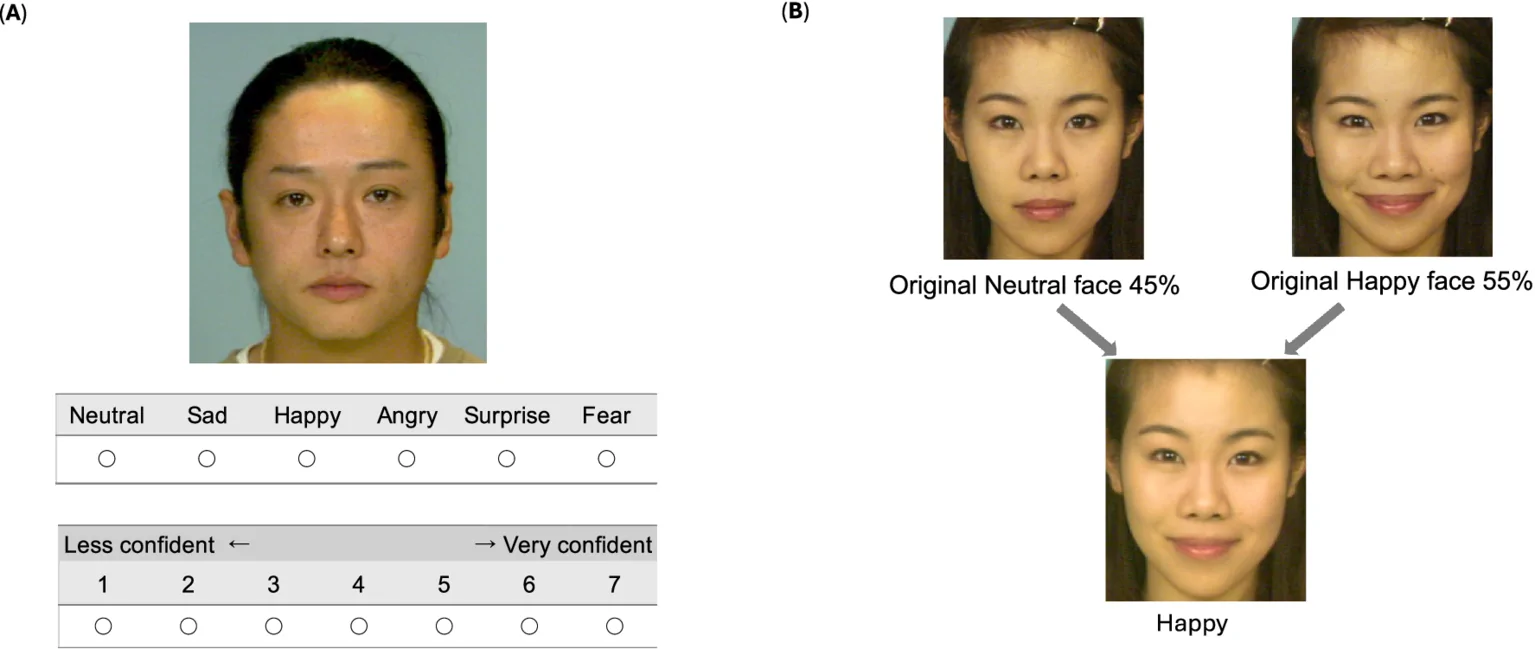
**(A)** Example of the emotion recognition display. **(B)** Schematic of the morphing procedure. Neutral and happy faces in the database were morphed in a given ratio to create happy facial expressions.

Figure 1A shows an example of an emotion recognition display. In each trial, a single facial image depicting one of the six emotional expressions (neutral, sad, happy, angry, surprised, or fearful) was presented. Participants selected the label that best described the expression from the six response options displayed below. Following each emotion recognition judgment, the participants rated their confidence on a 7-point scale (1 = very low confidence, 7 = very high confidence). For each expression category, the participants completed four trials with images from four different individuals, for a total of 24 trials. The presentation order of different facial expression images and the four individuals was intermixed, and each participant performed the task in a random order. The recognition accuracy for each expression was calculated as the mean accuracy across the four trials. The overall expression recognition score was computed as the average accuracy across all six expression categories.

#### 2.2.2 Generation of facial expressions

Six facial expressions were used: neutral, happy, sad, surprised, angry, and fearful. These categories represent commonly studied emotional expressions in facial emotion recognition research. A neutral expression was included because recognizing the absence of emotional signals is important for everyday social interaction (Mill et al., 2009). Expressions of disgust were not included because prior research has suggested that the recognition of disgust is less stable and more variable compared with other basic emotions (Russell, 1994). In addition, the identification of disgust expressions has been reported to be relatively less consistent in Japanese samples (Matsumoto, 1992), which may introduce additional variability in performance measures.

Stimuli were created using images from two validated facial expression databases: the ATR and AIST facial expression databases (Fujimura and Umemura, 2018). Twelve models (six men and six women) were selected. For each emotional category, stimuli consisted of two male and two female faces. Each model contributed two emotional conditions to balance identity across expressions.

To control and standardize the emotional intensity across categories, facial expressions were generated using a morphing procedure implemented in FantaMorph 5. As shown in Figure 1B, emotional stimuli were created by blending a neutral face with its corresponding emotional expression in predefined ratios. For example, a stimulus defined as 45% neutral/55% happy consisted of 45% neutral facial information and 55% happy expression, thereby specifying the intensity of the emotional signal.

Morphing ratios were determined with reference to the discrimination thresholds reported in previous morph-based emotion recognition studies (Kumada et al., 2011). The final ratios were set as follows: sad (45% neutral/55% sad), happy (45% neutral/55% happy), angry (45% neutral/55% angry), surprised (60% neutral/40% surprised), and fearful (35% neutral/65% fearful). Neutral faces were presented as unmorphed originals (100% neutral), without any blending with emotional expressions.

To reduce ceiling effects and minimize reliance on diagnostic cues, images depicting open mouths with visible teeth were excluded from the happy and angry expressions. Instead, closed-mouth expressions were used. These procedures were implemented to equate task difficulty across emotional categories and to enhance sensitivity to subtle age- and gender-related differences.

#### 2.2.3 PES

The PES comprises 20 items across two subscales: cognitive empathy (10 items) and affective empathy (10 items). The PES has demonstrated good internal consistency and construct validity for both cognitive and affective empathy in previous studies (Brett et al., 2023; Ye et al., 2024).

The scale was translated into Japanese using a forward-backward translation procedure. Semantic equivalence with the original English version was confirmed and the final Japanese version was used in the present study. Participants responded to each item using a 5-point Likert scale (1 = rarely, 2 = occasionally, 3 = about half the time, 4 = almost always, and 5 = always).

### 2.3 Data exclusion criteria

Participant details are shown in Table 1. Before the analysis, the data were screened based on predefined exclusion criteria to ensure response validity and data quality. First, participants with extremely short (<180 s) or long (>1,800 s) completion times were excluded. This resulted in the removal of 101 participants, leaving 847 participants for further analysis. Second, response time outliers were identified within each age × gender group using the interquartile range (IQR) method. Values falling outside 1.5 times the IQR were excluded, resulting in the removal of an additional 61 participants (remaining N = 786). Third, uniform response patterns were examined in the PES question set. These cases were considered unlikely to reflect meaningful task engagement. For each participant, the number of unique response values across the 20 items was calculated. Participants who used only a single response value across all PES items were classified as inattentive responders and were excluded. This procedure removed 90 participants (remaining *N* = 696). Finally, uniform response patterns were also examined in the emotion recognition. Only one participant met this criterion and was excluded. After applying the predefined exclusion criteria, the final sample comprised 101 women in their 30s, 119 women in their 50s, 122 women in their 70s, 90 men in their 30s, 118 men in their 50s, and 145 men in their 70s, yielding a total of 695 participants.

### 2.4 Statistical analysis

All statistical analyses were conducted using IBM SPSS (version 30.0). The significance level was set at *α* = 0.05 (two-tailed). Effect sizes are reported as partial eta squared (η_p_^2^) for analyses of variance (ANOVAs) and odds ratios (ORs) for the mixed-effects logistic regression models. To control the familywise error rate across the multiple ANOVAs, the Holm–Bonferroni sequential procedure (Holm, 1979) was applied separately to two families of tests. The first family comprised the primary age-related effect from each of the seven emotion recognition accuracy ANOVAs: the main effect of age group (overall accuracy, sad, surprise, angry, and happy expressions), the main effect of gender (fear expressions), or the age × gender interaction (neutral expressions), as applicable. The second family comprised the main effect of gender tested separately for cognitive and affective empathy (two tests), corrected jointly because both address the same underlying question—whether men and women differ in empathy—applied to two related subscales. Effects are considered significant only if they survived Holm correction (α = 0.05). Further details of the correction are provided in Supplementary Table S1.

#### 2.4.1 Emotion recognition accuracy

To examine the effects of age and gender on emotion recognition performance, two-way ANOVAs were conducted with age group (30s, 50s, and 70s) and gender (men and women) as between-subject factors. First, the total recognition accuracy averaged across all emotion categories was analysed. Subsequently, separate two-way ANOVAs were conducted for each emotion category to assess emotion-specific age and gender effects. *Post hoc* comparisons were conducted to control for multiple testing when significant main effects or interactions were observed.

#### 2.4.2 Confidence indices

To characterize the metacognitive patterns, two indices were computed: the high-confidence error rate (HCER) and high-confidence correct rate (HCCR). The HCER was defined as the proportion of incorrect trials accompanied by high confidence (*P* [high confidence | incorrect]). The HCCR was defined as the proportion of correct trials accompanied by high confidence (*P* [high confidence | correct]). High-confidence responses were operationally defined as confidence ratings of 6 or 7 on the 7-point scale. For descriptive purposes, the HCER and HCCR were calculated for each age × gender group.

Because each participant contributed multiple trials (up to 24), trial-level observations within the same participant are not independent. For inferential analyses, we therefore used mixed-effects logistic regression (Generalized Linear Mixed Model; SPSS 30.0 GENLINMIXED procedure) with a binomial distribution and logit link function, conducted separately for incorrect and correct trials. In each model, the dependent variable was whether the response was accompanied by high confidence (1 = confidence ≥6, 0 = confidence ≤5). Age group (30s, 50s, 70s; reference category: 30s) and gender (men, women; reference category: women)were entered as categorical fixed effects, consistent with the decade-based recruitment design, and a random intercept for participant was included to account for the within-participant correlation of repeated trials. Intraclass correlation coefficients (ICCs) were computed using the standard latent-variable-scale formula for logistic mixed models, ICC = σ^2^/(σ^2^ + π^2^/3), where σ^2^ is the estimated variance of the random intercept. Two participants who responded correctly on all trials contributed no error trials and were excluded from the HCER analysis (*N* = 693); all 695 participants were included in the HCCR analysis.

#### 2.4.3 Empathy and its relation to emotion recognition

The internal consistency of the Japanese version of the PES was assessed separately for the cognitive and affective subscales using Cronbach’s alpha. Subscale scores for cognitive and affective empathy were computed and their sum served as the overall empathy score. To examine the association between empathy and emotion recognition ability, Pearson’s correlation analyses were conducted between the empathy scores (cognitive, affective, and total) and recognition accuracy. Where appropriate, the correlations were computed separately for each age and gender group.

## 3 Results

### 3.1 Emotion recognition performance

#### 3.1.1 Overall accuracy

**Figure 2:**
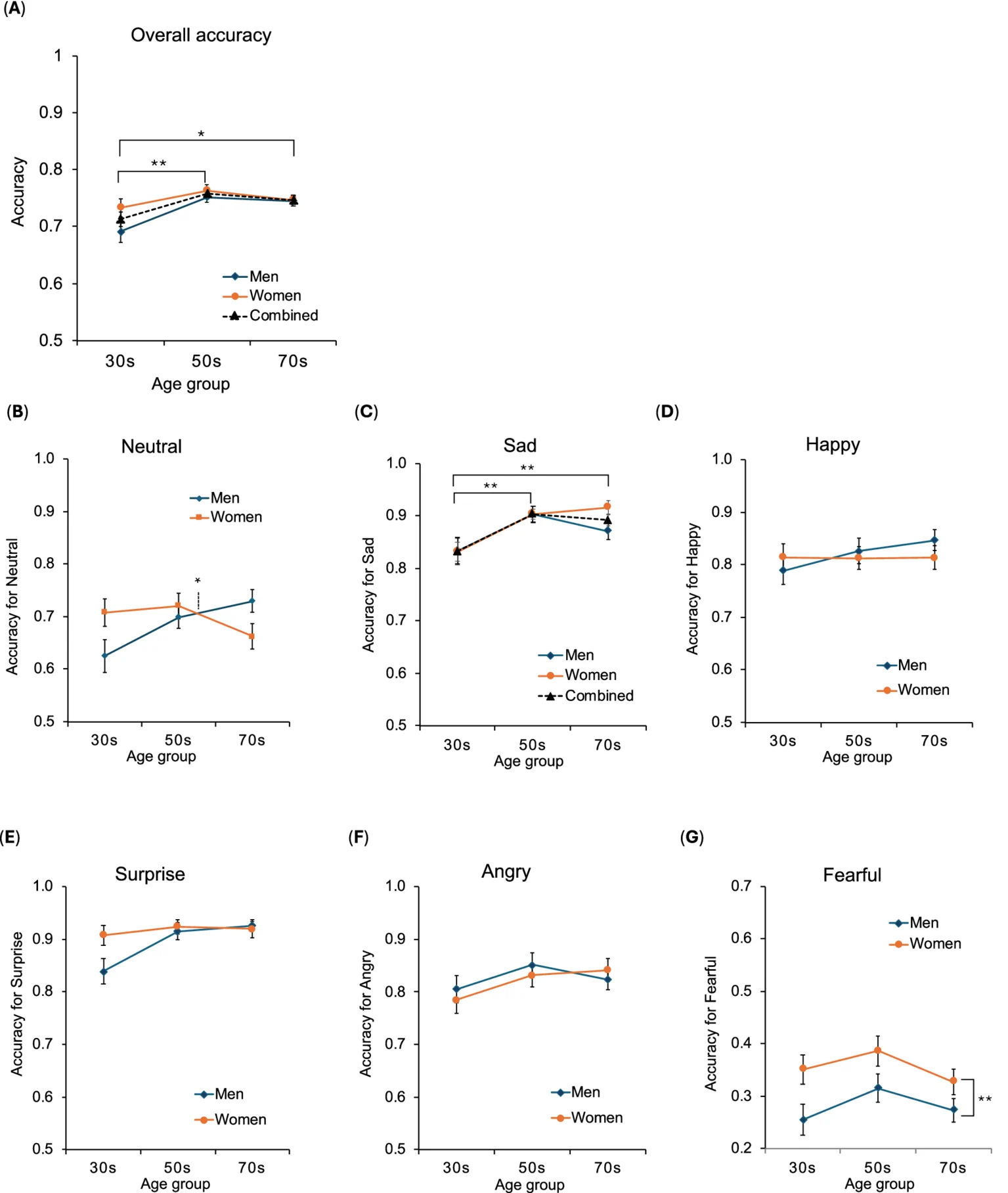
**(A)** Overall emotion recognition accuracy across age groups. **(B–G)** Emotion recognition accuracy for each emotion category. Error bars represent ±1 standard error of the mean (*SEM*). Lines for men and women are shown descriptively; a combined (gender-averaged) line is shown where only the main effect of age group was significant. Asterisks indicate, as applicable: Significant *post hoc* comparisons between age groups collapsed across gender **(A,C)**; the significant age × gender interaction **(B)**; or the significant main effect of gender, indicated by the bracket between men and women **(G)**. No significant effects survived Holm correction for panels **D–F**. The *y*-axis range in panel **(G)** differs from other panels to better display the accuracy range for fearful expressions. *p < 0.05, **p < 0.01 asterisks in panels **A** and **C** denote Bonferroni-corrected post hoc comparisons; asterisks in panels **B** and **G** denote Holm-corrected omnibus effects (the age × gender interaction and main effect of gender, respectively).

Figure 2A shows the overall emotion recognition accuracy in each age group. Higher values indicate better emotion recognition performance. A two-way between-subjects ANOVA was conducted on the overall accuracy rates with age group (30s, 50s, 70s) and gender (men, women) as the between-subjects factors. The analysis revealed a significant main effect of age group *F*(2, 689) = 6.71, *p* = 0.001, η_p_^2^ = 0.019. Neither the main effect of gender, *F*(1, 689) = 2.40, *p* = 0.122, η_p_^2^ = 0.003, nor the age × gender interaction, *F*(2, 689) = 1.63, *p* = 0.197, η_p_^2^ = 0.005 was significant. Bonferroni-corrected comparisons showed that the 50s group scored significantly higher than the 30s group, *p* < 0.001, and the 70s scored significantly higher than the 30s group, *p* = 0.026. These results indicate higher accuracy in the 50s and 70s groups, which did not differ significantly from each other, compared with the 30s group.

#### 3.1.2 Emotion-specific accuracy

Figures 2B–G shows the emotion recognition accuracy for each emotion category across age groups. As shown in the figure, the effect of age group on emotion recognition appears to vary by emotion type. A two-way between-subjects ANOVA was conducted on emotion recognition accuracy, with age group (30s, 50s, 70s) and gender (men, women) as between-subjects factors.

**Figure 3:**
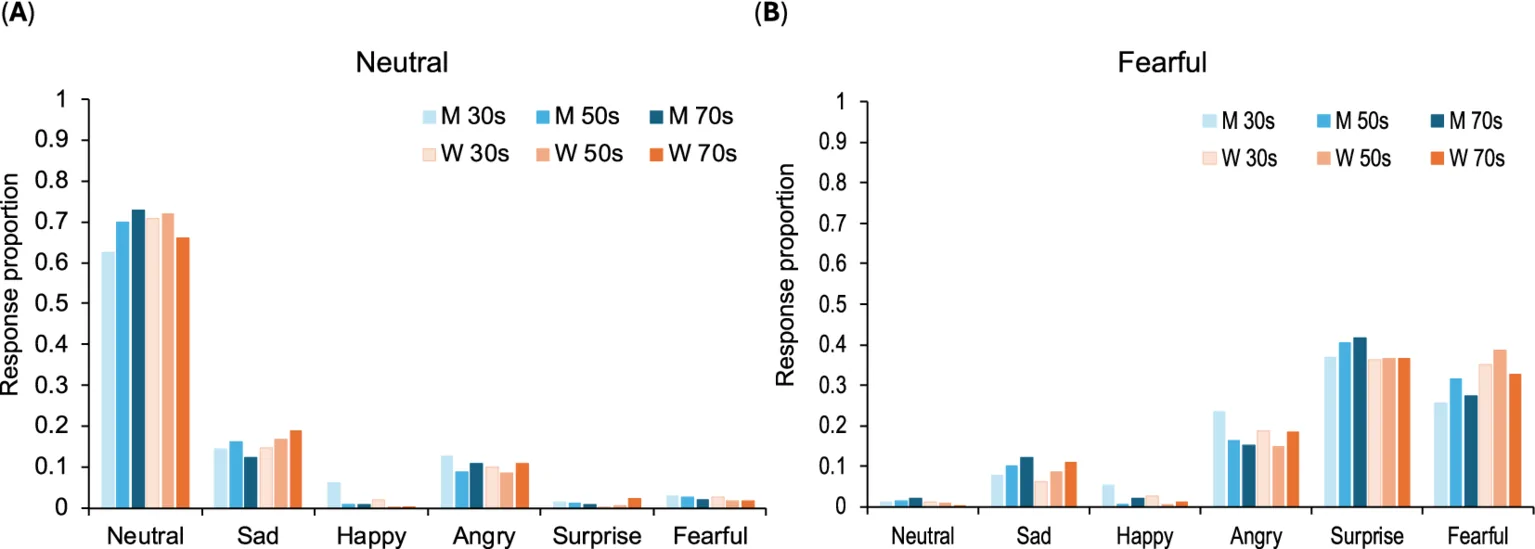
Distribution of response labels selected for **(A)** Neutral expressions and **(B)** Fearful expressions, shown separately for men (M) and women (W) in each age group (30s, 50s, 70s). Each bar represents the mean proportion of trials on which each response label was selected; bars over “Neutral” in **(A)** and “Fearful” in **(B)** indicate correct responses, and all other bars indicate misclassifications.

For neutral expressions, a two-way between-subjects ANOVA revealed a significant age × gender interaction, *F*(2, 689) = 4.72, *p* = 0.009, η_p_^2^ = 0.014. Neither the main effect of age group, *F*(2, 689) = 1.35, *p* = 0.261, η_p_^2^ = 0.001, nor that of gender, *F*(1, 689) = 0.03, *p* = 0.870, η_p_^2^ < 0.001, was significant. To further examine this interaction, *post hoc* comparisons were conducted. Within men, Bonferroni-corrected comparisons indicated a significant difference between the 30s and 70s groups (*p* = 0.010), with higher accuracy in the 70s group. No significant age group differences were observed in women. When examining gender differences within each age group, significant differences were observed in the 30s (*p* = 0.030) and 70s (*p* = 0.037), but not in the 50s. In the 30s, women showed higher accuracy than men, whereas in the 70s, men showed higher accuracy than women. These results indicate that age-group differences in neutral expression recognition differ between men and women. To examine which expressions participants selected when their responses were incorrect, we inspected the distribution of response labels for each expression category. Figure 3A shows the distribution of response labels. In all groups, misclassifications of neutral faces were biased toward negative expressions (sad and angry), with women in their 70s showing the strongest tendency to label neutral faces as sad.

For sad expressions, a main effect of age group was found, *F*(2, 689) = 8.18, *p* < 0.001, η_p_^2^ = 0.023, indicating age group differences in emotion recognition. Bonferroni-corrected comparisons indicated significant differences between the 30s and 50s groups (*p* < 0.001) and between the 30s and 70s groups (*p* < 0.001), indicating that accuracy in the 50s and 70s was higher than in the 30s.

For fearful expressions, a main effect of gender was found, *F*(1, 689) = 11.25, *p* = 0.001, η_p_^2^ = 0.016, indicating higher accuracy in women. The main effect of age group was not significant, *F*(2, 689) = 2.42, *p* = 0.089, η_p_^2^ = 0.007. The age × gender interaction was not significant, *F*(2, 689) = 0.17, *p* = 0.845, η_p_^2^ < 0.001. The results indicate consistently higher performance in women, while the effect of age showed only a marginal trend that did not reach statistical significance. The distribution of response labels was examined in the same manner for fearful expressions (Figure 3B). Men tended to misclassify fear as surprise across all age groups, whereas women selected the correct label more frequently, consistent with the observed gender main effect.

For surprise expressions, the main effect of age did not survive the Holm correction for multiple comparisons, *F*(2, 689) = 3.19, uncorrected *p* = 0.042, Holm-corrected *p* = 0.126, η_p_^2^ = 0.009. Neither the main effect of gender, *F*(1, 689) = 1.22, *p* = 0.27, η_p_^2^ = 0.002, nor the age × gender interaction, *F*(2, 689) = 1.96, *p* = 0.14, η_p_^2^ = 0.006, was significant.

For happy expressions, neither the main effect of age, *F*(2, 689) = 0.79, *p* = 0.456, η_p_^2^ = 0.002, nor that of gender, *F*(1, 689) = 0.38, *p* = 0.537, η_p_^2^ = 0.001, nor the age × gender interaction, *F*(2, 689) = 0.75, *p* = 0.470, η_p_^2^ = 0.002, was significant.

For angry expressions, the main effect of age approached significance, *F*(2, 689) = 2.48, *p* = 0.084, η_p_^2^ = 0.007, but neither the main effect of gender, *F*(1, 689) = 0.16, *p* = 0.687, η_p_^2^ < 0.001, nor the age × gender interaction, *F*(2, 689) = 0.55, *p* = 0.579, η_p_^2^ = 0.002, was significant.

### 3.2 Confidence in emotion recognition

**Figure 4:**
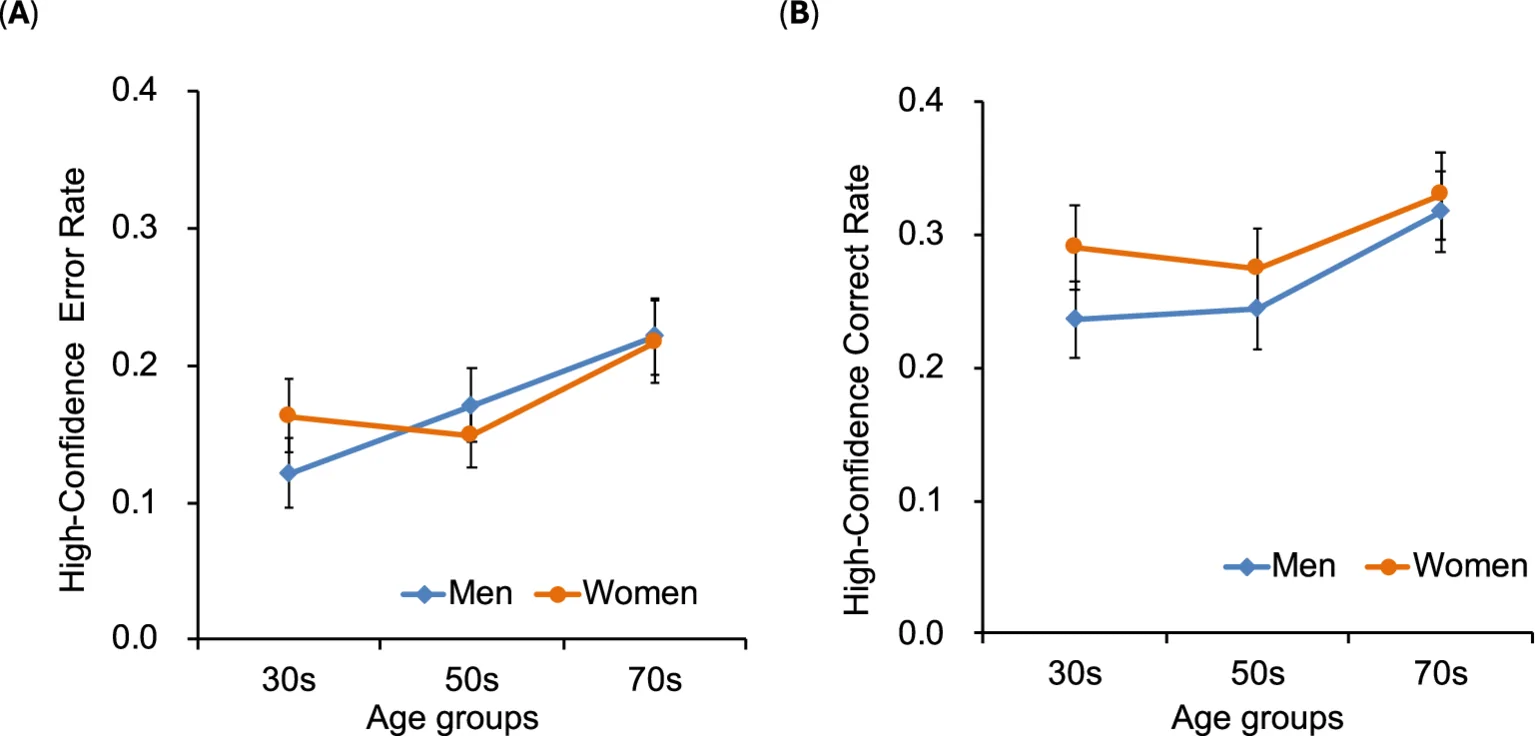
Age group differences in high-confidence responses, with **(A)** high-confidence error rate (HCER) and **(B)** high-confidence correct rate (HCCR) across age groups. Men and women are plotted separately. A mixed-effects logistic regression revealed a significant effect of age group on HCER (*p* = 0.015) but not on HCCR (*p* = 0.060). Gender was not a significant predictor in either analysis. Error bars represent ±1 SE based on participant-level proportions.

To examine age-group differences in confidence during emotion recognition, we analyzed high-confidence responses separately for incorrect and correct trials across all emotions using mixed-effects logistic regression (see Section 2.5.2)To assess whether the effect of age group on high-confidence responding varied between incorrect and correct trials, a combined mixed-effects logistic regression was conducted on all trials (*N* = 16,680). The model included response correctness (correct versus incorrect) and its interaction with age group as fixed effects, as well as a random intercept for participant. The descriptive results are shown in Figure 4.

#### 3.2.1 High-confidence error rate (HCER)

A mixed-effects logistic regression with a random intercept for participant was conducted on incorrect trials to examine whether the probability of high-confidence errors varied by age group and gender. Only incorrect trials were included, and the dependent variable was whether the incorrect response was accompanied by high confidence. Two participants who responded correctly on all 24 trials contributed no error trials and were excluded from this analysis (*N* = 693, 4,321 trials). The intraclass correlation coefficient (ICC = 0.50) indicated that 50% of the variance in high-confidence error responses was attributable to between-participant differences, confirming that accounting for the non-independence of trials within participants was necessary.

The effect of age group on HCER was significant, *F*(2, 4,317) = 4.23, *p* = 0.015. Compared to the 30s group (reference), the 70s group was significantly more likely to produce high-confidence errors (OR = 1.89, 95% CI [1.21, 2.96], *p* = 0.006), indicating that adults in their 70s were approximately 1.9 times more likely to commit high-confidence errors than those in their 30s (Figure 4A). The difference between the 50s and 30s groups did not reach statistical significance (OR = 1.25, 95% CI [0.78, 1.99], *p* = 0.358). Gender was not a significant predictor of HCER, *F*(1, 4,317) = 0.025, *p* = 0.875 (OR = 0.97, 95% CI [0.68, 1.39]). The variance of the random intercept was σ^2^ = 3.26, *p* < 0.001.

#### 3.2.2 High-confidence correct rate (HCCR)

A mixed-effects logistic regression with a random intercept for participant was conducted on correct trials to evaluate whether high-confidence correct responses were predicted by age group and gender (*N* = 695, 12,359 trials). The intraclass correlation coefficient (ICC = 0.63) indicated that 63% of the variance in high-confidence correct responses was attributable to between-participant differences.

Neither age group, *F*(2, 12,355) = 2.82, *p* = 0.060, nor gender, *F*(1, 12,355) = 0.916, *p* = 0.339, was a significant predictor of HCCR. Relative to the 30s group, neither the 70s (OR = 1.39, 95% CI [0.85, 2.26], *p* = 0.188) nor the 50s (OR = 0.80, 95% CI [0.48, 1.32], *p* = 0.376) differed significantly in the likelihood of high-confidence correct responses. Gender was not a significant predictor of HCCR, *F*(1, 12,355) = 0.92, *p* = 0.339 (OR = 0.83, 95% CI [0.56, 1.22]). The variance of the random intercept was σ^2^ = 5.70, *p* < 0.001.

#### 3.2.3 Test of the age group × correctness interaction

To formally assess whether the effect of age group on high-confidence responding differed between incorrect and correct trials, a combined mixed-effects logistic regression was conducted on all trials (*N* = 16,680). The model included age group, gender, response correctness, and the age group by correctness interaction as fixed effects, with a random intercept for participant. The age group by correctness interaction was not significant, *F*(2, 16,673) = 0.28, *p* = 0.756 (σ^2^ = 6.11, ICC = 0.65). Although age group significantly predicted high-confidence error rates (HCER) but not high-confidence correct response rates (HCCR) when each was tested separately, this pattern did not reflect a statistically confirmed difference in the effect of age group between the two response types. Therefore, the HCER and HCCR findings are reported as two separate results: a significant age-group effect on high-confidence errors, and no significant age-group effect on high-confidence correct responses.

Together, these results indicate age group differences in confidence: aging was associated with a greater likelihood of expressing high confidence when incorrect. Overall, the confidence analyses indicate an age-group effect on high-confidence errors, without evidence of a corresponding effect on high-confidence correct responses.

### 3.3 PES score

#### 3.3.1 Reliability of the Japanese version of PES

Internal consistency of the Japanese version of the PES was assessed using Cronbach’s alpha. The cognitive empathy subscale showed excellent reliability (*α* = 0.95), and the affective empathy subscale showed good reliability (α = 0.88). The overall scale also showed high reliability (α = 0.93). Across age groups, Cronbach’s alpha for cognitive empathy was 0.93, 0.96, and 0.94 for the 30s, 50s, and 70s groups, respectively. For affective empathy, the values were 0.87, 0.90, and 0.88. These results indicate that the Japanese version of the PES has stable and adequate reliability across age groups.

#### 3.3.2 Effect of gender and age on cognitive and affective empathy

**Figure 5:**
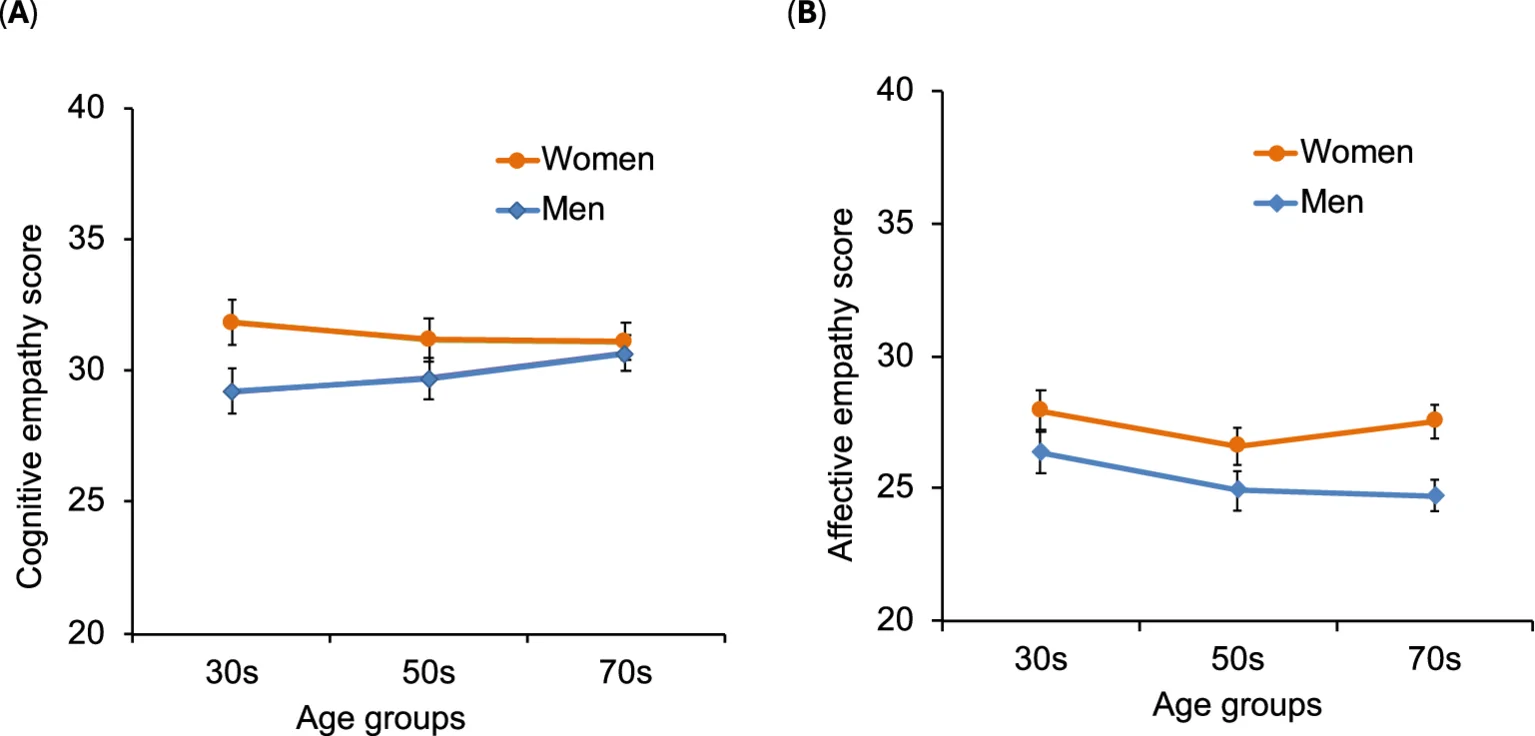
Empathy scores across age groups, with **(A,B)** cognitive empathy and **(B)** affective empathy. Error bars represent ±1 standard error of the mean (SEM).

Figure 5 shows cognitive and affective empathy scores across age groups. To examine the effects of gender and age on these scores, two-way ANOVAs were conducted separately for cognitive and affective empathy, with gender (men, women) and age group (30s, 50s, 70s) as between-subjects factors. For cognitive empathy, the analysis revealed a significant main effect of gender, *F*(1, 689) = 4.58, *p* = 0.033, η_p_^2^ = 0.007, which remained significant after Holm-Bonferroni correction (*p*_Holm_ = 0.033), indicating that women scored higher than men overall. The main effect of age group, *F*(2, 689) = 0.15, *p* = 0.85, η_p_^2^ < 0.001, and the gender × age interaction, *F*(2, 689) = 0.967, *p* = 0.38, η_p_^2^ = 0.002, were not significant. Exploratory *post hoc* comparisons using the Bonferroni correction showed that women in their 30s scored significantly higher than men (*p* = 0.032), whereas no significant gender difference was observed in the 50s age group (*p* = 0.189) and in the 70s age group (*p* = 0.652). However, these findings should be interpreted with caution, given the absence of a significant gender × age interaction.

For affective empathy, the two-way ANOVA revealed a significant main effect of gender, *F*(1, 689) = 13.99, *p* < 0.001, η_p_^2^ = 0.020, with women scoring higher than men. The main effect of age group, *F*(2, 689) = 2.29, *p* = 0.10, η_p_^2^ = 0.007, and the gender × age interaction, *F*(2, 689) = 0.206, *p* = 0.81, η_p_^2^ < 0.001, were not significant. *Post hoc* comparisons using Bonferroni correction were performed. No significant gender differences were observed in the 30s (*p* = 0.143) or 50s (*p* = 0.082). However, in the 70s group, a significant gender difference was observed (*p* = 0.002), with women scoring higher than men.

Overall, women scored higher than men on both cognitive and affective empathy. Although the overall patterns were broadly similar across age groups, the descriptive trends suggested that gender differences in cognitive empathy may decline with age, whereas those in affective empathy appeared relatively stable. However, these patterns should be interpreted cautiously, given the absence of significant interaction effects.

Correlation analyses revealed significant positive associations between cognitive and affective empathy across all age and gender groups (*r* = 0.56–0.74, all *p* < 0.001). When the correlations were examined by age group, the magnitudes of the associations were highly similar (30s: *r* = 0.64; 50s: *r* = 0.61; 70s: *r* = 0.63; all *p* < 0.001). The results suggest that the structural association between cognitive and affective empathy remains stable across adulthood and gender.

#### 3.3.3 Relationship between emotional face recognition and empathy

The relationship between the accuracy of emotional facial recognition and empathy was examined. Overall, accuracy showed a weak but statistically significant positive correlation with cognitive empathy (*r* = 0.13, *p* < 0.001). By contrast, no significant association was observed with affective empathy (*r* = 0.06, *p* = 0.12). These results suggest that facial emotion recognition accuracy may be more closely associated with cognitive empathy, although the overall effect size was small (*r* = 0.13). Correlation analyses were conducted separately for men and women in their 30s, 50s, and 70s to examine whether the relationship between facial emotion recognition and empathy differed by age and gender. The results are presented in Table 2. Specifically, associations between facial emotion recognition scores and affective empathy scores, as well as between facial emotion recognition scores and cognitive empathy scores, were analyzed within each subgroup. No significant correlations were found among women across all age groups or among men in their 30s or 50s. By contrast, significant positive correlations were observed only among men in their 70s. In this group, facial emotion recognition scores were significantly correlated with cognitive empathy (*r* = 0.25, *p* = 0.002) and affective empathy (*r* = 0.20, *p* = 0.015). Holm–Bonferroni correction was applied within each empathy subscale family. The correlation with cognitive empathy remained significant after correction (*r* = 0.25, Holm-corrected *p* = 0.012), whereas the correlation with affective empathy did not (*r* = 0.20, uncorrected *p* = 0.015, not significant after correction). Scatterplots of these associations for each age × gender group are provided in Supplementary Figure S1.

**Table 2:** Correlations between emotion recognition accuracy and empathy across age and gender groups.

Group		*N*	Cognitive empathy		Affective empathy	
Gender	Age		*r*	*p*	*r*	*p*
Men	30s	90	0.09	0.407	0.02	0.884
	50s	118	0.11	0.224	0.10	0.275
	70s	145	**0.25**	0.002**	0.20	0.015
Women	30s	101	0.08	0.401	0.04	0.726
	50s	119	0.06	0.496	0.07	0.444
	70s	122	0.15	0.097	0.11	0.229

*p* values shown are uncorrected. Asterisks indicate significance after Holm–Bonferroni correction, applied separately within each empathy subscale (cognitive empathy: 6 comparisons; affective empathy: 6 comparisons). **p < 0.01 (Holm-corrected). Bold values indicate statistically significant correlations (*p* < .05).

To determine whether the association between cognitive empathy and recognition accuracy in men in their 70s was driven by a specific emotion, exploratory correlations were computed between cognitive empathy and accuracy for each of the six expressions. None of the six correlations reached significance after Holm–Bonferroni correction (all corrected *p* ≥ 0.054), although fearful expressions showed the strongest association (*r* = 0.22, uncorrected *p* = 0.009, Holm-corrected *p* = 0.054). These exploratory, subgroup-level analyses should be interpreted cautiously given the reduced sample size and are presented descriptively rather than inferentially.

## 4 Discussion

This study examined the development of emotional face recognition from middle to late adulthood. To delineate this trajectory, cross-sectional differences were assessed among adults in their 30s, 50s, and 70s, extending beyond conventional young–old comparisons. Adults in their 70s did not demonstrate significantly lower accuracy than those in their 30s across overall accuracy and all six emotion categories. When significant age-group differences emerged, accuracy was higher in the 70s, particularly in overall accuracy, neutral expressions among men, and sad expressions. This finding contrasts with the widely reported pattern that older adults exhibit lower facial emotion recognition accuracy than younger adults (Olderbak et al., 2019; Ruffman et al., 2008), which is typically based on comparisons between young adults and adults aged 60 years or older. The results reveal age-group differences in facial emotion recognition and confidence judgments across adulthood. The observed pattern does not support a simple monotonic decline. If these cross-sectional differences reflect underlying developmental change, they suggest that emotion recognition performance is shaped by the interaction between accumulated perceptual experience and changes in metacognitive monitoring, although cohort-based explanations remain plausible.The summary of the main findings is as follows. First, the overall emotion recognition accuracy did not show a simple age-related decline. Instead, accuracy was higher in 50s and 70s than in the 30s. Moreover, age-group effects differed by gender and emotional category, indicating that age group differences were not uniform across emotions. In particular, men’s recognition of neutral expressions differed among age groups while women’s remained stable, resulting in a reversal of the gender advantage between the 30s and 70s, whereas women consistently outperformed men in fear recognition across age groups. Second, age group differences were associated with an increase in high-confidence errors, without evidence of a corresponding effect on high-confidence correct responses. This pattern suggests that age group differences in emotion recognition extend beyond perceptual accuracy to metacognitive monitoring. Third, women scored higher than men on both cognitive and affective empathy, although the magnitude of gender differences varied across age groups. The association between emotional recognition and empathy was generally weak, with significant associations observed only among men in their 70s with cognitive empathy. Taken together, these findings suggest that facial emotion recognition across adulthood follows emotion-specific and gender-dependent patterns, and that age group differences extended not only to perceptual accuracy but also the metacognitive evaluation of performance.

### 4.1 Emotion-specific differences

Our findings indicate that age-group differences in facial emotion recognition are emotion-specific and gender-modulated. This pattern is consistent with prior meta-analytic and empirical evidence demonstrating that aging effects vary across emotional categories, rather than reflecting a uniform decline (Isaacowitz and Stanley, 2011; Ruffman et al., 2008).

For neutral expressions, a significant age × gender interaction was observed: women showed higher accuracy than men in their 30s, while men outperformed women in their 70s. This crossover effect was primarily attributable to increased accuracy among men across age groups, rather than a decline among women, whose performance remained relatively stable. As neutral expressions lack clear diagnostic cues, their recognition appears to depend more on integrative or context-dependent processing (Mill et al., 2009; Orgeta and Phillips, 2008), which may increase susceptibility to cohort-related experiential differences between men and women. A possible, albeit speculative, explanation involves cohort differences in occupational history. In current cohorts of individuals in their 50s and 70s, labor force participation diverged markedly by gender: men were predominantly full-time employees, while a substantial proportion of women in these generations were homemakers who did not engage in continuous paid employment (Brinton, 1993). Sustained occupational exposure to ambiguous social and emotional cues, such as interpreting supervisors’, colleagues’, or clients’ non-explicit facial reactions, may foster more accurate interpretation of ambiguous expressions. This is consistent with evidence that facial expression recognition can be shaped by domain-specific experience (Conson et al., 2013) and that accumulated social-role experience across the lifespan can enhance sensitivity to specific emotional cues (Lin et al., 2024). This account predicts both a relative advantage among men in their 70s and a corresponding bias among women toward negative interpretation of ambiguous cues. Supporting this prediction, analysis of misclassification patterns (Figure 3A) indicated that incorrect responses to neutral faces were biased toward negative expressions (sad and angry) across all groups, with women in their 70s exhibiting the strongest tendency to label neutral faces as sad. However, this account does not explain the female advantage observed in the 30s, which is more plausibly attributed to a general female advantage in processing socially salient emotional cues, as reported elsewhere in the literature (Kret and De Gelder, 2012; McClure, 2000). It is important to note that the occupational-exposure account represents only one plausible interpretation among several cohort-based explanations (see also Limitations).

For sad expressions, a main effect of age group indicated higher recognition accuracy among middle-aged and older adults compared to younger adults. This pattern aligns with either a developmental account, in which recognition of certain negative emotions improves or is maintained from middle adulthood onward due to increased social experience or accumulated exposure to emotional situations, or a cohort-based account, in which generational differences in socialization or task familiarity produce similar outcomes. In both cases, the observed benefit appears to reflect accumulated experience rather than an artifact of task difficulty, as a comparable age-group effect was not observed for fear or anger, despite these expressions being calibrated to a similar level of difficulty using the same morphing procedure. This selectivity suggests that, if accumulated experience underlies the age-group difference in sad-expression recognition, the benefit may be specific to sadness rather than to negative emotions more broadly. However, the present cross-sectional design cannot distinguish between developmental and cohort-based explanations, nor can it clarify why such a benefit, if genuine, would be selective to sadness. This distinction could potentially be addressed within a cross-sectional framework by directly assessing occupational history and social-role experience alongside emotion recognition, rather than relying on age group as a proxy for accumulated experience; however, such measures were not collected in the present study.

For fear expressions, the female advantage was robust across all age groups. In contrast to findings for neutral and sad expressions, the effect of age group did not reach significance, indicating that age-related differences in fear recognition were not evident in this sample. This result is consistent with previous research demonstrating that women outperform men in recognizing threat-related or socially salient negative emotions (Kret and De Gelder, 2012; McClure, 2000). The non-significant age by gender interaction further suggests that the female advantage is stable across age groups. Analysis of response-label distributions (Figure 3B) clarified the nature of this gender difference: men consistently misclassified fearful expressions as surprise across age groups, while women more frequently selected the correct label. The perceptual similarity between fear and surprise in upper-face configuration, attributed to shared action units involving the eyebrows and eyelids (Roy-Charland et al., 2014), may contribute to this pattern. Therefore, the observed female advantage may reflect more precise discrimination between visually similar expressions, rather than a general difference in fear sensitivity.

By contrast, no significant effects were observed for surprise, happiness, or anger under the present task conditions. This selective pattern is consistent with evidence that aging effects in emotion recognition are not global but depend on stimulus characteristics, emotional category, and task demands (Isaacowitz and Stanley, 2011).

Importantly, although previous research has documented both age-group differences and gender differences in emotion recognition, relatively few studies have directly examined how these factors interact across the adult lifespan. The present findings contribute to this gap by demonstrating that age-group differences are not only emotion-specific but also gender-dependent, particularly for ambiguous expressions such as neutral faces.

Together, these findings support the view that age-group differences in facial emotion recognition are emotion-dependent and gender-modulated, rather than reflecting a generalized age-related decline.

### 4.2 Confidence and metacognitive interpretation

An important finding of the present study is that age group was associated with an increased likelihood of high-confidence errors, whereas no corresponding association was found for high-confidence correct responses. However, a direct test of the age group × correctness interaction did not reach significance (Section 3.2.3). We therefore interpret the present findings with appropriate caution, reporting the age-group effect on HCER as an independent finding rather than as evidence that older adults show a confidence pattern selectively tied to incorrect responses.

This result aligns with previous research indicating age-related changes in metacognitive monitoring and calibration. Studies of memory and perceptual decision-making have reported that older adults may show reduced accuracy in monitoring their performance, particularly overconfidence in errors (Dodson et al., 2007). Similarly, research on confidence calibration suggests that aging can be associated with diminished correspondence between subjective confidence and objective accuracy (Overhoff et al., 2021; Palmer et al., 2014).

The present findings extend the literature to the field of facial emotion recognition. While age-related differences in emotion recognition accuracy are well documented, and metacognition of emotion recognition has been investigated (Kelly and Metcalfe, 2011; Bègue et al., 2019), limited research has explored how metacognitive evaluation of emotion judgments varies across age groups. The increase in high-confidence errors observed in the present study suggests that aging may affect not only perceptual or interpretive processes but also the monitoring of socioemotional decisions. Moreover, because high-confidence correct responses showed no significant age-group differences, the findings do not support a substantial general shift toward elevated subjective certainty. The age-group effect observed for high-confidence errors should therefore be interpreted as an independent finding, consistent with the caveat above. Taken together, these findings suggest that age-related differences in facial emotion recognition involve first- and second-order evaluative processes. Therefore, incorporating confidence measures provides insights into socioemotional aging that would not be captured by accuracy indices alone.

### 4.3 Empathy across age and its relation to emotion recognition

This study found that women scored higher than men on both cognitive and affective empathy. However, the gender difference in cognitive empathy was relatively small and close to the multiple-comparison threshold, whereas the gender difference in affective empathy was more robust. This pattern is consistent with extensive prior research indicating that gender differences are typically larger for emotional responsiveness than for cognitive perspective-taking (Christov-Moore et al., 2014; Eisenberg and Lennon, 1983).

In contrast to some previous findings suggesting age-related changes in empathy (e.g., Grühn et al., 2008; O’Brien et al., 2013), including a recent meta-analysis reporting a small but significant increase in emotional empathy across adulthood (Jarvis et al., 2024), the present study found no significant main effect of age group for either cognitive or affective empathy. Furthermore, no statistically significant age × gender interactions were observed, indicating that gender differences in empathy were relatively stable across adulthood.

The overall correlations between empathy and facial emotion recognition were weak, with only a small positive association observed between cognitive empathy and recognition accuracy. This modest relationship is consistent with previous research indicating that the link between empathy and emotion recognition ability is generally small and variable (Murphy and Lilienfeld, 2019; Olderbak and Wilhelm, 2017), suggesting that dispositional empathy and the perceptual discrimination of facial emotions represent partially distinct processes. Although emotion recognition relies on perceptual sensitivity and interpretive accuracy, self-reported empathy reflects broader motivational and interpersonal dispositions, which may not translate directly into performance-level accuracy.

Significant associations between only cognitive empathy and emotion recognition were observed only among men in their 70s. Although this subgroup finding should be interpreted cautiously, it may indicate that, in later adulthood, particularly among men, individual differences in cognitive empathy become more closely aligned with socioemotional perceptual performance. One possibility is that cognitive empathy plays a supportive or compensatory role in age-related cognitive changes, which are most pronounced in the 70s (Reuter-Lorenz and Park, 2014). Consistent with this account, a similar but non-significant positive association was observed among women in their 70s (*r* = 0.15), indicating that the pattern may not be exclusive to men. However, because this association did not reach statistical significance, this interpretation requires caution and further investigation.

Overall, these findings suggest that although the structural organization of empathy remains stable across adulthood, its mean-level expression and its relationship with emotion recognition vary as a function of age and gender and may be shaped by broader contextual factors.

### 4.4 Methodological strengths

This study was designed with several methodological strengths. First, unlike many previous studies that have primarily compared young adults, often university students, with older adults aged 60 years and above, this study examined facial emotion recognition across three adult life stages: the 30s, 50s, and 70s. By including middle-aged participants, we moved beyond a dichotomous comparison. We were able to characterize age-group differences across adulthood more continuously, rather than assuming a uniform decline. Second, the stimulus difficulty was carefully calibrated to avoid ceiling effects and enhance sensitivity to individual and age-related differences. By equating task demands across emotional categories, the design allowed for more precise detection of subtle age-group and gender-specific patterns. Finally, by incorporating confidence measures, the study extends beyond accuracy to capture the metacognitive aspects of emotion recognition, revealing age group differences in high-confidence errors that would not have been detectable from performance alone. Together, these methodological features improve the interpretability of the findings and provide a comprehensive account of facial emotion recognition across adulthood.

### 4.5 Limitations and future directions

This study has several limitations that should be acknowledged. First, the present study employed a cross-sectional design. Although age group differences were observed among the 30s, 50s, and 70s groups, cross-sectional comparisons cannot distinguish developmental changes from cohort effects. Differences between age groups may reflect generational variations in socialization, educational background, or cultural norms rather than individual aging processes. Additionally, adults in their 70s who participate in commercial online panels and complete smartphone-based tasks likely represent a highly selected, technologically engaged subgroup of their generation. This selection may further confound cohort-related factors with aging effects. Consequently, the observed age-group differences cannot be directly interpreted as evidence of developmental trajectories, and the findings should be considered in light of this limitation.

Second, data were collected through a web-based survey. Although online sampling enabled the recruitment of a relatively large, demographically diverse adult sample, the experimental conditions were less tightly controlled than those in laboratory settings. The stimulus presentation time was not fixed, and the participants completed the task using their own devices under varying environmental conditions. These factors may have introduced variabilities in attention, viewing distance, and perceptual clarity. Furthermore, participants were recruited through a commercial online panel, and no screening for mental health or neurological disorders was implemented. Therefore, the potential influence of these conditions on emotion recognition performance cannot be excluded. Future studies employing controlled laboratory paradigms or standardized presentation conditions would help validate and extend the present findings.

Third, although task difficulty was intended to be equated across neutral and emotional expressions using morphed stimuli, the accuracy results suggest that difficulty was not fully matched across emotion categories. In particular, fear expressions were relatively more difficult to recognize. Because task difficulty can modulate both perceptual sensitivity and decision strategies, this imbalance may have contributed to the observed age and gender effects. Future research should employ more precise psychometric calibration procedures to ensure equivalent levels of discriminability across emotional categories.

Fourth, the emotion recognition task included a limited number of trials per expression category. Although this design reduced participant burden and was appropriate for an online format, a larger number of trials would enable more precise estimation of individual differences and improve reliability at the emotion-specific level.

Finally, this study focuses on static facial stimuli. Real-world emotion perception often involves dynamic expressions and contextual cues. Future research should examine whether the observed age- and gender-related patterns generalize to more ecologically valid settings.

## 5 Conclusion

In conclusion, the present study provides evidence of age group differences in facial emotion recognition across adulthood, accompanied by age group differences in confidence judgments. While the cross-sectional design precludes direct conclusions about developmental trajectories, the findings of reduced accuracy in individuals in their 30s, comparable performance in the 50s and 70s, and anincreased likelihood of high-confidence errors among older adults establish a preliminary framework for understanding changes in emotional face recognition from middle to late adulthood. This pattern generates specific, testable hypotheses regarding developmental change that require confirmation through longitudinal research. These results underscore the importance of examining midlife alongside older adulthood and considering both accuracy and confidence when evaluating age-related differences in emotion recognition.

## Data Availability

The datasets presented in this study can be found in online repositories. The names of the repository/repositories and accession number(s) can be found at: The datasets analyzed for this study can be found in the OSF repository: https://osf.io/3wkbu/overview?view_only=f54df090f9654fbbad30c8e5c75bb351.
