# Supplementary material for: Age-related differences in facial emotion recognition across adulthood: accuracy and confidence in the 30s, 50s, and 70s
Source: Front Psychol. 2026 Sep 3;17:1859795. doi: 10.3389/fpsyg.2026.1859795 (PMC13581502; doi:10.3389/fpsyg.2026.1859795)
Supplement: Supplementary file 1 [file Data_Sheet_1.PDF]

Supplementary Materials

Age-Related Differences in Facial Emotion Recognition Across Adulthood: Accuracy and Confidence in the 30s, 50s, and 70s

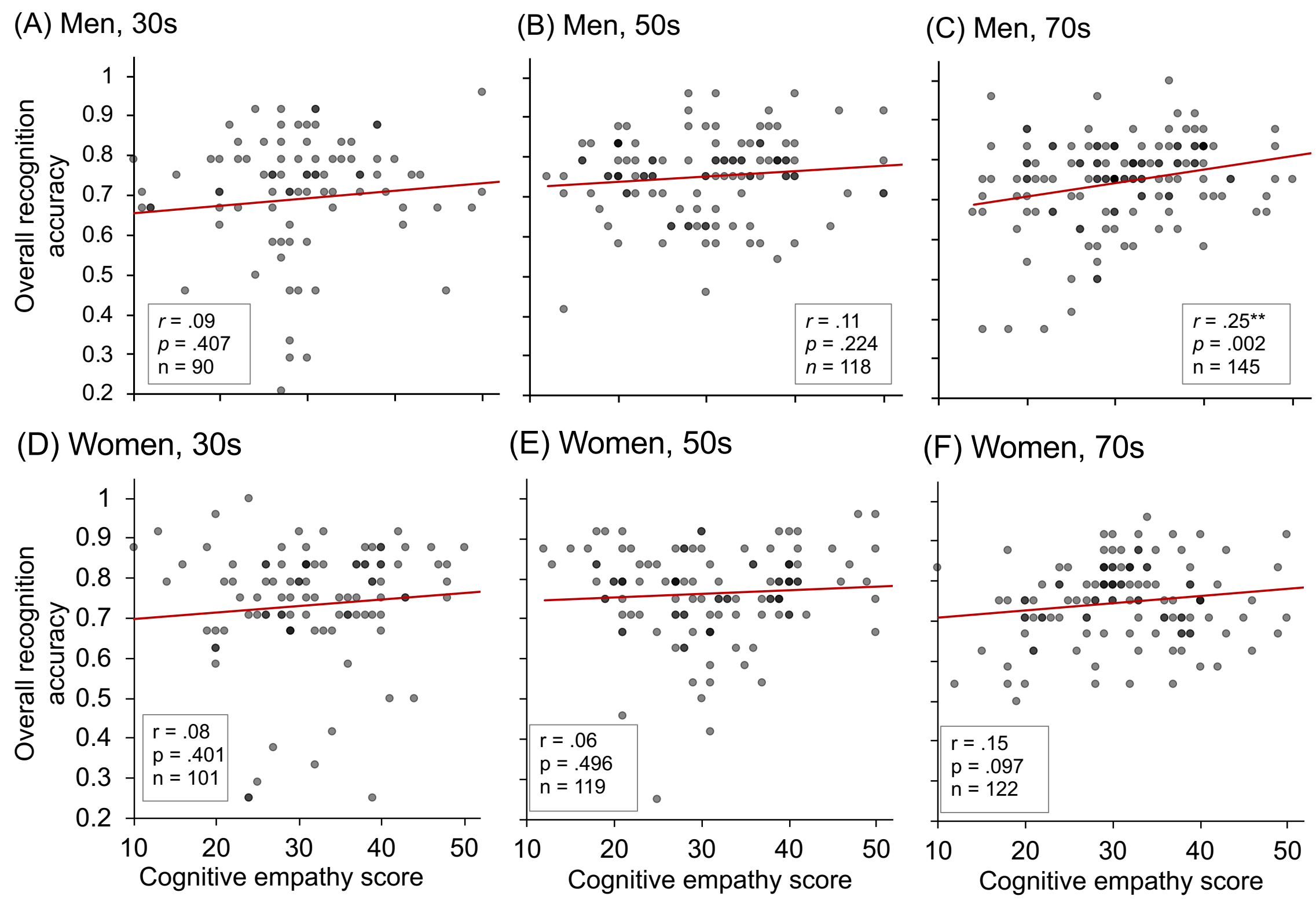

Figure S1. Scatterplots showing the association between cognitive empathy scores and overall facial emotion recognition accuracy, separately by age group and gender. Red lines indicate linear regression fits. A significant positive association was observed only among men in their 70s (Panel (C)).

Supplementary Table S1. Holm–Bonferroni correction applied within each family of tests.

Family	Test	Uncorrected p	Holm-corrected p
Accuracy (n=7)	Overall accuracy (age)	.001	.001
	Sad (age)	<.001	<.001
	Fear (gender)	.001	.001
	Neutral (age×gender)	.009	.036
	Surprise (age)	.042	.126
	Angry (age)	.084	.168
	Happy (age)	.456	.456
Empathy (n=2)	Cognitive Empathy (gender)	.033	.033
	Affective Empathy (gender)	<.001	<.001

Note. Holm–Bonferroni correction was applied separately within each family (accuracy: n = 7 tests; empathy gender effects [cognitive and affective empathy]: n = 2 tests), as described in Section 2.5.
